# Histone methyltransferase SMYD1: playing a crucial role in disease progression

**DOI:** 10.3389/fmolb.2026.1769660

**Published:** 2026-02-09

**Authors:** Lingqian Duan, Yang Lou, Kan Huang, Kailing Pan, Xianguo Chen

**Affiliations:** 1 School of Medicine, ShaoXing University, Shaoxing, China; 2 Department of Cardiothoracic Surgery, Affiliated Jinhua Hospital of Zhejiang University School of Medicine, Jinhua, China

**Keywords:** cancer, cardiovascular diseases, disease progression, histone methyltransferase, SMYD1

## Abstract

Histone methyltransferase SET and MYND domain-containing 1 (SMYD1), a member of the SMYD family, catalyzes the methylation of lysine residues on histone proteins. This modification is pivotal in regulating chromatin structure and gene expression, influencing processes such as cell proliferation, differentiation, and development. Primarily expressed in muscle tissues, SMYD1 plays a crucial role in muscle development and function. However, accumulating evidence suggests its involvement in the progression of various diseases, including cancer, cardiovascular diseases, and metabolic disorders. By modulating key signaling pathways and gene expression profiles, SMYD1 affects cellular processes such as cell cycle regulation, apoptosis, and inflammation. This review aims to explore the multifaceted roles of SMYD1 in disease progression, highlighting its potential as a therapeutic target. Understanding the molecular mechanisms underlying the effects of SMYD1 will be essential for developing strategies to manipulate its activity for disease prevention and treatment.

## Introduction

Histone methyltransferases (HMTs) are critical enzymes involved in modulating gene expression and transcription through post-translational modifications of histones (Fields et al., 2023). These modifications participate in various biological processes and are associated with numerous diseases, including cancer (Wu et al., 2025). The SET- and MYND-domain containing (SMYD) proteins, a subfamily of HMTs, have attracted significant attention in recent years.

The SMYD family is defined as a group of lysine methyltransferases, distinguished by the presence of a methyltransferase SET domain and a zinc finger-containing MYND domain. Notably, the catalytic SET domain in SMYD proteins is interrupted by the MYND domain (Rubio-Tomás, 2021). SMYD family members play diverse roles in cellular processes, including the regulation of gene expression, DNA damage response, and cell proliferation (Han et al., 2024; Shi et al., 2024; Lepore Signorile et al., 2023). Furthermore, individual SMYD family members exhibit distinct tissue-specific expression patterns and functions. For example, SMYD2 has been implicated in pulmonaryhypertension by methylating non-histone proteins such as PPARγ, which accelerates the proliferation of pulmonary arterial smooth muscle cells and contributes to pulmonary hypertension (Li et al., 2024). SMYD3 is frequently overexpressed in leukemia stem cells and has been shown to stimulate fatty acid β-oxidation in a methyltransferase activity-dependent manner (Zhou et al., 2025).

SMYD1, a protein specific to cardiac and skeletal muscle, functions as an HMT and regulates downstream gene transcription, playing crucial roles in cellular processes, particularly in the development of cardiac and skeletal muscle (Tang et al., 2024; Cicatiello et al., 2024). Understanding its functions, regulatory mechanisms, and implications in diseases may provide valuable insights into disease pathogenesis and therapeutic opportunities. This review focuses on SMYD1, covering its general characteristics, discovery, molecular mechanisms, roles in disease, inhibitors, and future research directions.

## SMYD1 structure and function

SMYD1 is a histone lysine methyltransferase specific to striated muscle, first identified in 1995 within the reverse reading frame of the murine CD8b gene (Hwang and Gottlieb, 1995). Located on the short arm of chromosome two at band 11.2 (2p11.2), the *SMYD1* gene encodes a protein of 490 amino acids, which is localized in both the nucleus and cytoplasm. SMYD1 displays tissue-specific expression, being confined to fetal and adult cardiac and skeletal muscle tissues (Rueda-Robles et al., 2021). In humans, the *SMYD1* locus encodes a single protein isoform. In contrast, the murine *Smyd1* gene undergoes alternative splicing, producing three distinct protein variants: Smyd1a and Smyd1b, which retain muscle-specific expression profiles, and Smyd1c, which is exclusively expressed in T lymphocytes.

Like other SMYD family members, SMYD1 contains two essential conserved structural domains: a SET lysine methyltransferase domain (130–140 amino acids) and a conserved zinc finger MYND domain at the N-terminus (residues 1-28), which includes seven cysteine residues and one histidine residue forming a C_4_-C_2_HC consensus sequence (Wang et al., 2021). The SET domain is interrupted by the MYND domain, resulting in two segments: the S-sequence and the core SET domain. The S-sequence, a small region, may facilitate cofactor binding or protein–protein interactions in association with the MYND domain (Sirinupong et al., 2010; Sims et al., 2002). Additionally, the SET domain is linked to the post-SET, SET-I, and pre-SET regions, all of which contribute to cofactor binding, substrate binding, and the structural stability of the protein (Couture et al., 2005; Wilson et al., 2002). The MYND domain, a zinc finger motif, is known for its ability to bind proline-rich regions, serving as a module for protein–protein interactions (Liu et al., 2007). In SMYD proteins, the MYND domain resides in the *N*-terminal lobe, where it interacts with the catalytic SET domain but does not engage in substrate or cofactor binding. Notably, deletion of the MYND domain does not affect methyltransferase activity *in vitro*, suggesting it is not essential for methylation (Abu-Farha et al., 2008). The C-terminal domain (CTD) structure is highly conserved across the SMYD family, with the primary difference being the extended and protruding αN helix in SMYD1, a feature unique to this protein, as SMYD2 and SMYD3 have shorter αN helices. The CTD appears to play a critical role in substrate binding, as the deletion of the CTD from SMYD1 enhances histone H3 binding and methylation. This suggests that the CTD may exert steric effects that regulate substrate access to the active site (Sirinupong et al., 2010).

As an HMT, SMYD1 catalyzes the mono-, di-, and tri-methylation of histone H3 at lysine 4 (H3K4), thereby promoting downstream gene transcription. SMYD1-mediated trimethylation of H3K4 (H3K4me3) enhances the transcriptional activation of p*eroxisome proliferator-activated receptor gamma coactivator 1-alpha* (*PGC-1α*). In *Smyd1*-knockout (KO) mice, H3K4me3 enrichment at the *PGC-1α* locus is significantly reduced, and luciferase reporter assays confirm that SMYD1 directly induces *PGC-1α* transcriptional activation. Functional analyses reveal that *PGC-1α* overexpression partially rescues mitochondrial respiration deficits in *Smyd1*-KO cardiomyocytes, whereas *PGC-1α* knockout abolishes the enhanced respiratory capacity conferred by SMYD1 overexpression (Warren et al., 2018). Consistent with these findings, SMYD1 promotes mitochondrial bioenergetics by transcriptionally upregulating *optic atrophy 1* (*OPA1*), a key regulator of mitochondrial fusion and oxidative phosphorylation (Szulik et al., 2023). Furthermore, Smyd1 facilitates H3K4me3 at the *IL-6* promoter region 2, enhancing *IL-6* transcription and contributing to inflammatory responses (Shamloul et al., 2021). During early cardiogenesis, Smyd1 binds to the promoter regions of *ISL LIM Homeobox* 1 (*Isl1*) and *GSK3β*, inducing H3K4me3 and activating their expression, which is critical for cardiogenesis (Wang et al., 2021; Chang et al., 2024). Conversely, SMYD1 also exhibits transcriptional repressive functions. Smyd1-mediated di-methylation of histone H3 at lysine 4 (H3K4me2) suppresses apoptosis and pyroptosis in the murine C2C12 myoblast cell line by transcriptionally repressing *Purinergic receptor P2X, ligand-gated ion channel, 7* (*P2RX7*) (Li et al., 2023). Histone acetylation, facilitated by histone acetyltransferases (HATs), leads to chromatin relaxation and enhances transcription, whereas histone deacetylation, mediated by histone deacetylases (HDACs), results in chromatin condensation and gene silencing (Kablan et al., 2025). SMYD1 has been shown to recruit HDACs to mediate transcriptional repression (Phan et al., 2005). Notably, SMYD1 directly binds to the promoter regions of *TGFβ3* and *Nppa/ANF*, suppressing their expression independently of global or locus-specific H3K4me3 changes (Franklin et al., 2016). These findings highlight the dual role of SMYD1 as both a transcriptional activator and repressor, highlighting its complex regulatory role in development, metabolism, and disease.

In addition to histone modifications, SMYD1 modulates the activity of various non-histone proteins. A notable non-histone substrate of SMYD1 is Heat Shock Protein 90 (Hsp90), a molecular chaperone involved in protein folding and stabilization. SMYD1 methylates Hsp90, enhancing its chaperone function, which is critical for maintaining cellular homeostasis under stress conditions (Jiang et al., 2011). Moreover, SMYD1 modulates the methylation of myocyte enhancer factor 2 (MEF2), promoting muscle-specific gene expression and contributing to muscle growth and repair (Zhu et al., 2023). SMYD1 also regulates the transcription factor skeletal NAC (skNAC), which drives the transcription of myoglobin (MB), a gene encoding a muscle-specific globin protein that facilitates oxygen transport. Mechanistically, SMYD1 interacts with the PXLXP motif of skNAC through its conserved MYND domain, mediating methylation at residue K1975 within the C-terminal region of skNAC. This modification enhances MB transcriptional activation (Zhu et al., 2023). Similarly, Tribbles3 (TRB3), a stress-responsive factor, binds to SMYD1 through its far N-terminal PXLXP motif. SMYD1 directly methylates TRB3, converting it into a corepressor that collaborates with SMYD1 to inhibit cardiomyocyte proliferation (Rasmussen et al., 2015). These findings emphasize that SMYD1-mediated methylation acts as a key regulatory node in epigenetic signaling, with its dysregulation being strongly associated with various pathological conditions, including developmental abnormalities, metabolic dysfunction, and cardiovascular diseases.

### SMYD1 in cardiovascular development and disease

As a lysine methyltransferase containing SET and MYND domains, Smyd1 plays a crucial role in the development and functional maintenance of the cardiovascular system, with its abnormalities being closely linked to various cardiovascular diseases. Smyd1 generates two isoforms, Smyd1_tv1 and Smyd1_tv2, through alternative splicing (Xu et al., 2024). Among these, Smyd1_tv1 is predominantly expressed in the myocardium and is essential for sarcomere organization in cardiomyocytes and cardiac function, whereas Smyd1_tv2 is mainly localized in skeletal muscle and is dispensable for cardiac development (Xu et al., 2024). By localizing to the sarcomeric M-line and enhancing its binding to myosin chaperone proteins (such as Hsp90α1 and Unc45b), Smyd1_tv1 maintains sarcomeric integrity and normal mitochondrial organization in cardiomyocytes. Its deficiency leads to cardiac hypertrophy, sarcomeric disarray, and cardiac dysfunction. In contrast, zebrafish expressing only Smyd1_tv2 die during the larval stage due to cardiac defects (Xu et al., 2024).

Smyd1b deficiency impairs the recruitment and integration of secondary heart field progenitor cells, leading to failure of cardiac looping, expansion of cardiac jelly, and downregulation of key transcription factors such as gata4, gata5, and nkx2.5, thereby triggering congenital heart defects (Prill et al., 2025). In myocardial ischemic injury, overexpression of Smyd1a upregulates OPA1 expression, promoting mitochondrial cristae remodeling and the formation of respiratory chain supercomplexes. This process enhances mitochondrial respiratory efficiency, reduces infarct size, and decreases cardiomyocyte apoptosis, ultimately exerting a cardioprotective effect on cardiac function. Notably, Smyd1 expression is downregulated in the myocardium of patients with heart failure, whereas it remains at normal levels in control patients, highlighting its importance in myocardial repair (Szulik et al., 2023).

Furthermore, Smyd1 regulates sarcomere assembly and homeostasis by monomethylating lysine 35 (K35) on myosin heavy chain (MyHC). Loss of this modification leads to the degradation of MyHC via the ubiquitin-proteasome system, resulting in sarcomeric defects in both cardiac and skeletal muscles. This regulatory mechanism is conserved in human-induced pluripotent stem cell-derived cardiomyocytes (Diofano et al., 2025). Smyd1 also directly interacts with CHD4 to cooperatively repress gene programs related to glycolysis, hypoxic response, and angiogenesis, which is critical for maintaining normal cardiac development (Shi et al., 2024).

Mutations in the human SMYD1 gene are associated with hypertrophic cardiomyopathy, dilated cardiomyopathy, and biventricular heart failure, further confirming that abnormalities in Smyd1 are a critical pathogenic factor in cardiovascular diseases (Jiao et al., 2021).

### SMYD1 in cancer

**FIGURE 1:**
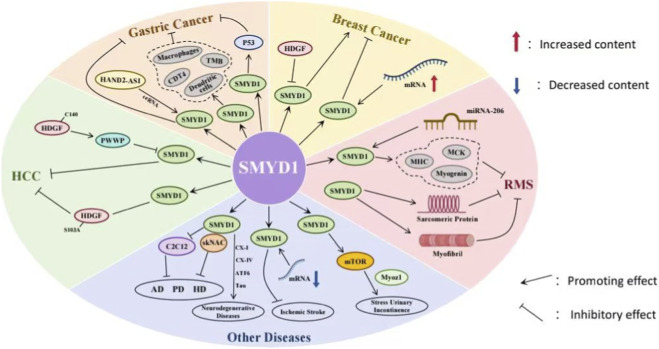
The regulatory roles of SMYD1 in cancer and other diseases. SMYD1 modulates several molecules and biological processes in various pathological contexts: In cancer (gastric cancer, breast cancer, hepatocellular carcinoma), SMYD1 interacts with factors such as HAND2 Antisense RNA 1 (HAND2-AS1), Hepatoma-Derived Growth Factor (HDGF), Pro-Trp-Trp-Pro domain (PWWP), and immune cells (macrophages, dendritic cells), influencing molecules like Tumor Protein 53 (P53), CD4-positive T cells (CD4^+^ T), and tumor mutational burden (TMB). In rhabdomyosarcoma (RMS), SMYD1 regulates muscle-related factors (Myogenic factor 4 (Myogenin), Myosin Heavy Chain (MHC), Muscle Creatine Kinase (MCK)) involved in sarcomeric protein synthesis and myofibril formation. In other diseases, SMYD1 interacts with skeletal muscle-specific Nuclear Factor Activated in T-cells (skNAC), NF-κB, C2C12 mouse myoblast cell line (C2C12), Cytochrome c Oxidase Subunits I (CX-I) and IV (CX-IV), Tau protein (Tau), and Activating Transcription Factor 6 (ATF6) in neurodegenerative diseases (Alzheimer’s disease (AD), Parkinson’s disease (PD), and Huntington’s disease (HD)). It also modulates mRNA expression in ischemic stroke and regulates mammalian target of rapamycin(mTOR) and myozenin 1(Myoz1) in stress urinary incontinence. SMYD1 exerts its functions by interacting with diverse partners, thereby influencing the progression of cancer, muscle-related diseases, and neurodegenerative diseases.

SMYD1, a member of the SMYD protein family with specific HMT activity, exhibits tumor-suppressive characteristics in various malignant tumors, including hepatocellular carcinoma (HCC), gastric cancer (GC), breast cancer (BC), and rhabdomyosarcoma (RMS) (Figure 1).

In HCC, SMYD1 has been identified as a potential tumor suppressor gene. Its expression is transcriptionally repressed by hepatoma-derived growth factor (HDGF). The mechanism involves HDGF recognizing and binding to the promoter region of the SMYD1 gene via its N-terminal PWWP domain, recruiting a transcriptional repressor complex, and leading to SMYD1 transcriptional silencing (Chen et al., 2018). Studies have shown that while the C-terminal 140-amino acid domain (C140) of HDGF does not directly bind to DNA, it significantly enhances the binding affinity of the PWWP domain to chromatin through conformational changes, resulting in an approximately 10-fold increase in the inhibitory efficiency of full-length HDGF compared to the PWWP domain alone (Chen et al., 2018). Functionally, downregulation of SMYD1 disrupts the transcriptional regulatory network of its downstream genes involved in the cell cycle and apoptosis, promoting proliferation, invasion, and metastasis in HCC cells (Yang and Everett, 2007; Yang and Everett, 2009). Additionally, the phosphorylation of serine 103 (S103) in HDGF is crucial for its ability to drive the malignant phenotype of HCC. Mutation of this site (S103A) does not affect its binding to the SMYD1 promoter but abrogates the oncogenic potential of HDGF, suggesting an auxiliary oncogenic mechanism independent of SMYD1 binding (Everett et al., 2011).

In GC, multiple studies have consistently demonstrated that the mRNA and protein expression levels of SMYD1 in GC tissues are significantly lower than in adjacent normal tissues, indicating its potential role as a tumor suppressor. Low SMYD1 expression is significantly associated with shorter overall survival (OS) and progression-free survival (PFS) in patients and serves as an independent risk factor for poor prognosis (Liu et al., 2023). At the molecular pathological level, SMYD1 expression correlates with the clinical stage of GC and is higher in patients with wild-type TP53, suggesting a potential interaction with the p53 signaling pathway (Liu et al., 2021). In the tumor microenvironment, SMYD1 expression is positively correlated with immune infiltration levels of CD4^+^ T cells, macrophages, and dendritic cells, and negatively correlated with tumor mutational burden (TMB), indicating that SMYD1 may affect the progression of GC by regulating anti-tumor immune responses (Liu et al., 2023). Its regulatory mechanism also extends to the epigenetic level; for example, the long non-coding RNA HAND2-AS1 can indirectly upregulate SMYD1 expression through the ceRNA mechanism, participating in the inhibitory pathway of GC (Liu et al., 2021).

In BC, SMYD1 also demonstrates distinct tumor-suppressive properties. Multiple bioinformatics analyses and experimental validations have shown that SMYD1 mRNA is consistently downregulated in various BC subtypes, including medullary carcinoma, ductal carcinoma, and lobular carcinoma, compared to normal breast tissues. This suggests that its inactivation is a common event in BC initiation (Song et al., 2019; Curtis et al., 2012). Patients with high SMYD1 mRNA expression exhibit significantly prolonged recurrence-free survival (RFS), with a hazard ratio (HR) of 0.73, indicating that SMYD1 may serve as a protective factor in BC (Györffy et al., 2010).

Regarding the regulatory mechanism, similar to HCC, SMYD1 expression in BC is inhibited by the oncogenic factor HDGF, suggesting a common regulatory axis across different cancer types (Chen et al., 2012). Notably, the mutation rate of the SMYD1 gene in BC samples is extremely low (approximately 3%), indicating that its downregulation is primarily driven by transcriptional and epigenetic mechanisms, rather than genetic mutations in its coding region (Gao et al., 2013; Cerami et al., 2012). During RMS differentiation, SMYD1 has also been identified as a key regulatory factor. As a muscle-specific HMT, SMYD1 is typically downregulated in RMS cells, and the restoration of its expression is closely linked to myogenic differentiation (Berkholz et al., 2024). miR-206 can promote RMS cell differentiation into mature myocytes by upregulating SMYD1 expression, which induces the expression of muscle-specific genes (e.g., Myogenin, MHC, MCK). While the loss of SMYD1 does not affect miR-206-mediated inhibition of proliferation, its expression is critical for the complete execution of the myogenic differentiation program. Additionally, SMYD1 is localized in both the nucleus and cytoplasm and may play a role in myofibrillogenesis by regulating the folding and assembly of sarcomeric proteins (Coda et al., 2015). Therefore, SMYD1 not only plays a pivotal role in the epigenetic reprogramming of RMS but may also serve as a potential therapeutic target for differentiation-inducing therapies.

### SMYD1 in other diseases

Recent studies have revealed an unexpected yet pivotal regulatory role of SMYD1 as a transcription factor in neurodegenerative diseases. Gene expression analysis in C2C12 myoblasts showed that following SMYD1 knockdown, transcripts associated with Alzheimer’s disease (AD), Parkinson’s disease (PD), and Huntington’s disease (HD)—including COX isoforms, NDUFA isoforms, ATPase isoforms, and the microtubule-associated protein Tau/MAPT—were significantly altered (Warren et al., 2018). Further investigations confirmed that SMYD1 and the transcription factor skNAC are co-expressed in the corticostriatal regions of both human and mouse brains, forming a heterodimeric complex in the subcortical regions of transgenic mouse models of AD, PD, and HD (Mayfield et al., 2020). These findings suggest that SMYD1 and skNAC may synergistically regulate neuro-related genes.

In experiments involving the reprogramming of C2C12 myoblasts into neurons, SMYD1 was identified as a rate-limiting factor in this differentiation process (Mayfield et al., 2020). Depletion of SMYD1 impaired the acquisition of neuronal phenotypes and significantly reduced dendritic density, while knockdown of skNAC had no such effect. Mechanistically, SMYD1 regulates key molecules involved in mitochondrial oxidative phosphorylation (e.g., subunits of Complex I and Complex IV), endoplasmic reticulum stress-related factors (e.g., ATF6), and Tau protein, contributing to essential neuronal processes such as energy metabolism and protein homeostasis. Dysfunction of SMYD1 may accelerate pathological changes, including mitochondrial dysfunction and abnormal protein aggregation, thus promoting the onset and progression of neurodegenerative diseases (Mayfield et al., 2020). The expression characteristics and potential role of SMYD1 in ischemic stroke have also been preliminarily explored. In a rat model of ischemic stroke induced by middle cerebral artery occlusion (MCAO), transcriptome sequencing and qRT-PCR validation revealed that SMYD1 mRNA expression was significantly downregulated in the brains of model rats compared to the sham-operated group, indicating that abnormal SMYD1 expression may be linked to the pathological progression of ischemic stroke (Ye et al., 2022). As a key regulatory gene involved in muscle differentiation and remodeling, Smyd1 plays an important role in the pathological mechanisms, treatment, and repair of stress urinary incontinence (SUI) (Fang et al., 2025). The core pathological features of SUI include urethral sphincter muscle fiber injury, neuromuscular atrophy, and urethral closure dysfunction, with insufficient muscle regeneration capacity being a critical factor in the persistence and recurrence of symptoms. Upregulation of Smyd1 directly promotes myogenic regeneration of the urethral sphincter, with its mechanism involving regulation of myofiber differentiation, stabilization of the myocyte cytoskeleton, and inhibition of proteolysis.

In SUI therapeutic strategies based on regenerative medicine, a thermosensitive hydrogel system containing leucine and decellularized extracellular matrix (dECM) has been shown to significantly upregulate Smyd1 expression by activating the mTOR signaling pathway. This system synergizes with muscle regeneration markers such as Myoz1, improving the structural integrity and contractile function of the urethral sphincter. In a female rat model of SUI, a composite injection system loaded with ZIF-8/PEG200@Mg nanoparticles for pretreating adipose-derived mesenchymal stem cells (ADSCs) achieved synchronous regeneration of both striated and smooth muscle fibers through sustained high expression of Smyd1. Simultaneously, it promoted the stability of neuromuscular junctions (NMJ), significantly increased leak point pressure (LPP), and restored urethral closure function. These findings confirm that targeted regulation of Smyd1 is a key mechanism for enhancing defective muscle regeneration of the urethral sphincter in SUI, providing molecular targets and experimental evidence for the development of novel SUI therapeutic regimens that simultaneously promote muscle regeneration and nerve repair (Fang et al., 2025).

### Regulation of SMYD1

To date, specific inhibitors targeting SMYD1 have not been successfully developed. However, accumulating evidence suggests that small-molecule compounds or therapeutic agents can modulate SMYD1-related signaling pathways, indirectly ameliorating pathological conditions associated with SMYD1 dysfunction. Preliminary investigations have also explored the regulatory effects of certain drugs on SMYD1. In the field of cardiac differentiation, Chang et al. (2024) reported that insulin and insulin-like growth factor 1 (IGF-1) effectively enhance cardiomyocyte differentiation efficiency in SMYD1-knockout cells. The underlying mechanism involves the reduction of p-ERK expression, a downstream molecule in SMYD1-deficient cells, rescuing cardiomyocyte differentiation defects. This finding highlights a potential molecular target for intervening in SMYD1 dysfunction-associated cardiac diseases.

Additionally, doxorubicin (DOX), an anthracycline drug commonly used in cancer treatment, was found to upregulate SMYD1 expression in human pluripotent stem cell-derived ventricular cardiomyocytes within 3D engineered cardiac tissues (Shamloul et al., 2021). SMYD1 overexpression subsequently regulates multiple critical processes, including cardiac gene expression, contractile function, calcium handling, and electrophysiological function. However, the dose-dependent cardiotoxicity of DOX limits its direct application in the treatment of SMYD1-related cardiac diseases.

In the broader context of SMYD family inhibitor research, several agents have been developed, such as AZ50552 targeting SMYD2 and BCI-121 targeting SMYD3. However, these inhibitors lack specific regulatory effects on SMYD1. Currently, research on SMYD1 is primarily focused on elucidating its biological functions and associated signaling pathways. No dedicated SMYD1 inhibitors have entered the development phase or clinical trials. The development of SMYD1-specific inhibitors will depend on further insights into its molecular structure and mechanism of action (P et al., 2023).

## Discussion and conclusion

As a lysine methyltransferase containing SET and MYND domains, SMYD1 possesses HMT activity and plays a crucial regulatory role in the physiological processes of multiple human tissues. Abnormalities in its function are closely linked to the onset and progression of various diseases (Zhu et al., 2023). Currently, no specific inhibitors targeting SMYD1 have been developed, and only a few indirect regulatory approaches are available (Berkholz et al., 2024). Regarding pathological mechanisms, SMYD1 dysfunction is associated with a wide range of diseases. In cardiovascular diseases, SMYD1 deficiency can lead to myocardial hypertrophy, congenital heart defects, and other conditions. Its expression is downregulated in the myocardium of patients with heart failure, and mutations in the SMYD1 gene are also linked to hypertrophic cardiomyopathy (Fan et al., 2019). However, overexpression of Smyd1a has been shown to protect cardiac function after ischemic injury. In neurological diseases, SMYD1 knockdown results in abnormal expression of transcripts associated with neurodegenerative diseases. Dysfunction of SMYD1 may exacerbate mitochondrial dysfunction, promoting the progression of diseases such as AD (Mayfield et al., 2020). Additionally, SMYD1 mRNA expression is significantly reduced in the brains of rats with ischemic stroke, suggesting its involvement in stroke-related pathology. In urinary system diseases, upregulated SMYD1 expression improves the structural integrity and contractile function of the urethral sphincter in SUI (Fang et al., 2025). In cancer, SMYD1 exhibits tumor-suppressive properties across various malignancies, including HCC and GC. Downregulation of SMYD1 expression promotes tumor cell proliferation, invasion, and other malignant behaviors, with low SMYD1 expression often correlating with poor prognosis in cancer patients (Han et al., 2024).

Regarding research on regulation and applications, no specific SMYD1 inhibitors have been developed to date. However, existing studies have found that insulin, IGF-1, and the anthracycline drug DOX can indirectly regulate SMYD1-related signaling pathways or its expression (Wang et al., 2010). Notably, DOX’s dose-dependent cardiotoxicity limits its direct clinical application. In the treatment of SUI, thermosensitive hydrogel systems and composite injection systems have shown therapeutic potential by upregulating SMYD1 expression (Chen et al., 2024). Additionally, in RMS, SMYD1 holds promise as a potential target for differentiation-inducing therapies. In conclusion, SMYD1 plays a key role in the pathogenesis and progression of various diseases, including cancer and cardiovascular disorders. It significantly influences treatment strategies and prognosis, positioning it as a promising therapeutic target for managing these diseases.
